# A rare and challenging case of intrapericardial hydatidosis

**DOI:** 10.1186/s13019-023-02455-3

**Published:** 2023-11-20

**Authors:** Fatmir Caushi, Emira Hysa, Ilir Skenduli, Lutfi Lisha, Alban Hatibi, Loreta Bica, Silvana Bala, Francesco Rulli

**Affiliations:** 1 Department of Thoracic Surgery, University Hospital “Shefqet Ndroqi”, Street “Shefqet Ndroqi”, 1001 Tirana, Albania; 2 Department of Surgery, “Our Lady of Good Counsel” University, Tirana, Albania

**Keywords:** Echinococcosis, Intrapericardial, Surgery

## Abstract

**Background:**

Hydatid cysts are most frequently located in the liver and lungs and very rarely can be found in the pericardium. Diagnosis and treatment are quite challenging, as the disease can present itself in many forms depending to the location and the complications that it might cause.

**Case presentation:**

A 22-year-old man presented to our hospital with ongoing dry cough for more than 1 month prior to admission. Other symptoms included chest pain, fatigue, low grade fever, and night sweats, which have worsened in the past 2 weeks. Physical examination revealed normal respiratory and heart function. Chest X-ray demonstrated mediastinal enlargement and left pleural effusion. Contrast-enhanced computed tomography images showed a walled cystic mass lesion measuring up to 56 × 50 mm in close proximity to the upper left atrium, ascending aorta and pulmonary artery, potentially localized in the pericardium, with a 10 mm endoatrial filling defect, findings were compatible with hydatid cyst, left pleural effusion and peripheral pulmonary upper left lobe consolidation. Cardiac involvement was excluded on magnetic resonance imaging and trans-esophageal ultrasound. The patient underwent fine needle aspiration of the affected lung and thoracocentesis. No malignancy was found, meanwhile the biopsy confirmed the presence of pulmonary infarction. In view of the imaging findings were highly suspicious of a hydatid cyst, we performed a test of antibody titers that was negative. The patient underwent left anterolateral thoracotomy, and after the opening of the pericardium, a cystic mass of 5 cm in diameter was found next to the left atrium and in close proximity with the left pulmonary veins. The content of the cyst was completely removed after the surgical area was isolated with gauze impregnated with hypertonic solution (NaCl 10%). The mass resulted to be an echinococcal cyst with multiple daughter cysts within it that did not penetrate/involve (perforate) the cardiac wall.

**Conclusion:**

Pericardial echinococcosis is a very rare pathology in which a high expertise multidisciplinary approach is required. The compression mass effect caused by the cyst can lead to complications, such as in our case where the pulmonary vein was compressed, leading to pulmonary infarction. The value of radiology studies and transoesophageal ultrasound are very important in the diagnosis. Surgery in these cases is always recommended, but preferred surgical approach is questionable. In cases such as ours, we recommend anterolateral thoracotomy.

## Background

Hydatid cysts are most frequently located in the liver and lungs and very rarely can be found in the pericardium [1]. Diagnosis and treatment are quite challenging, as the disease can present itself in many forms depending to the location and the complications that it might cause [2–5].

## Case presentation

A 22-year-old man presented to our hospital with ongoing dry cough for more than 1 month prior to admission. Other symptoms included chest pain, fatigue, low grade fever, and night sweats, which have worsened in the past 2 weeks. Physical examination revealed normal respiratory and heart function. Chest X-ray demonstrated mediastinal enlargement and left pleural effusion.

**Fig. 1:**
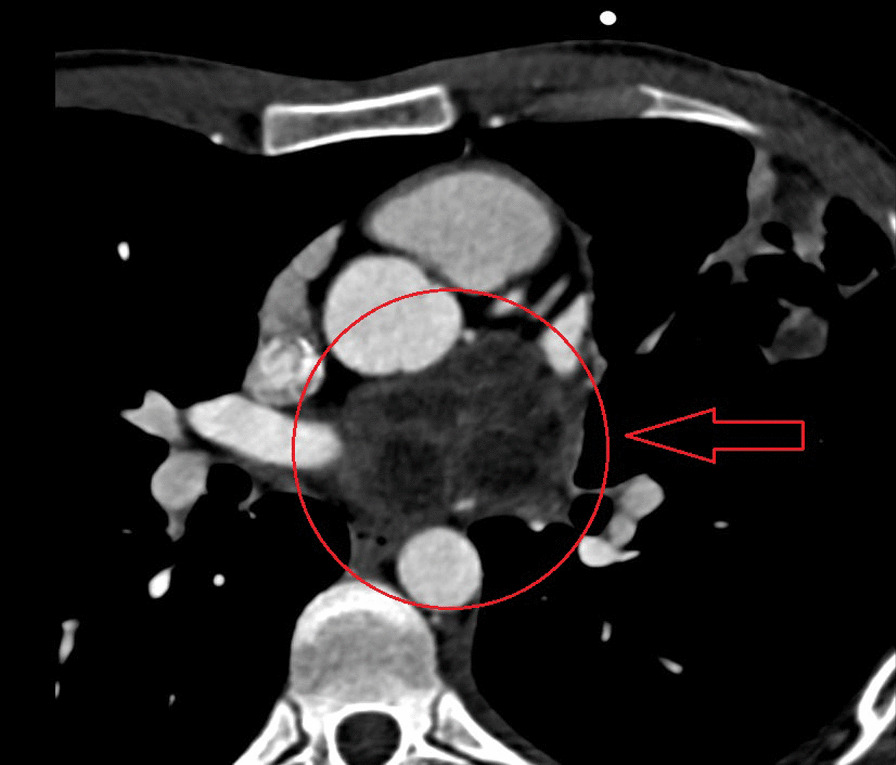
Retrocardiac multicystic mass on CECT

CECT images showed a walled cystic mass lesion measuring up to 56 × 50 mm in close proximity to the upper left atrium, ascending aorta and pulmonary artery, potentially localized in the pericardium, with a 10 mm endoatrial filling defect, findings were compatible with hydatid cyst, left pleural effusion and peripheral pulmonary upper left lobe consolidation (Fig. 1).

**Fig. 2:**
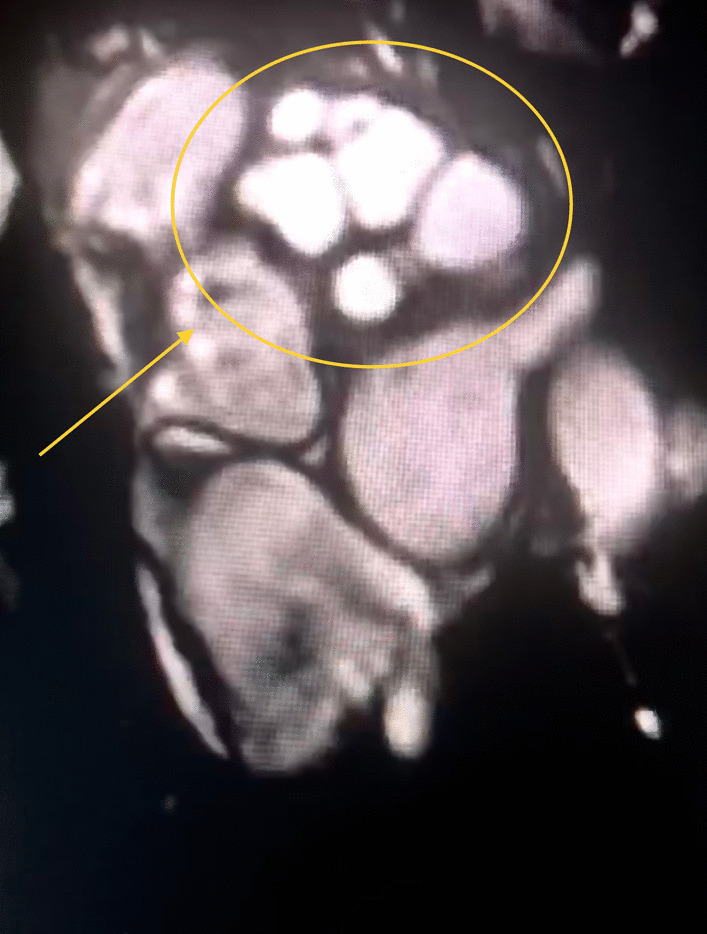
Hypointense multicameral cystic mass on MRI

**Fig. 3:**
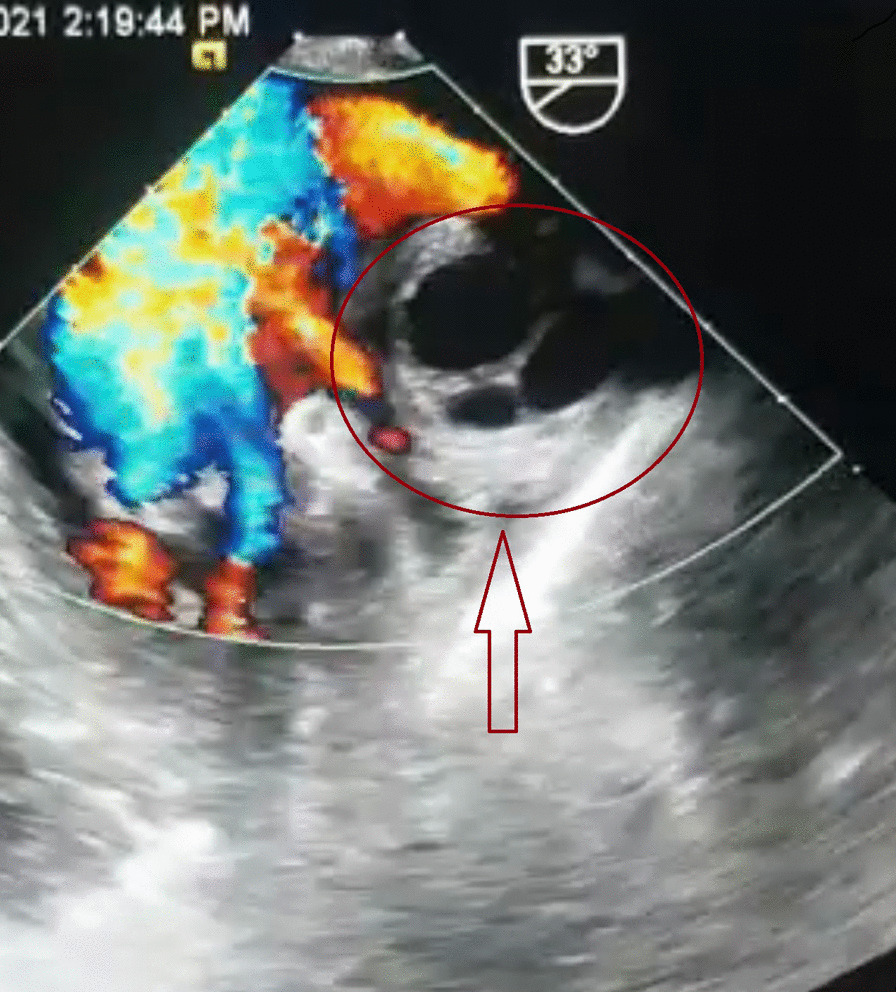
A Gharbi type II hydatid mass on transesophageal ultrasound

On MRI (Fig. 2) and transesophageal ultrasound (Fig. 3), an extracardiac multicameral cystic mass adjacent to the left atrium was observed, with a mass effect in the left upper pulmonary vein with no cardiac infiltration after contrast medium.

The patient underwent FNA of the upper left lobe consolidation and thoracocentesis for the cytological evaluation of the fluid. No malignancy was seen, while the biopsy evidenced the presence of pulmonary infarction.

As far as the imaging findings suspected a hydatid cyst, a test of antibody titers was performed and was negative.

**Fig. 4:**
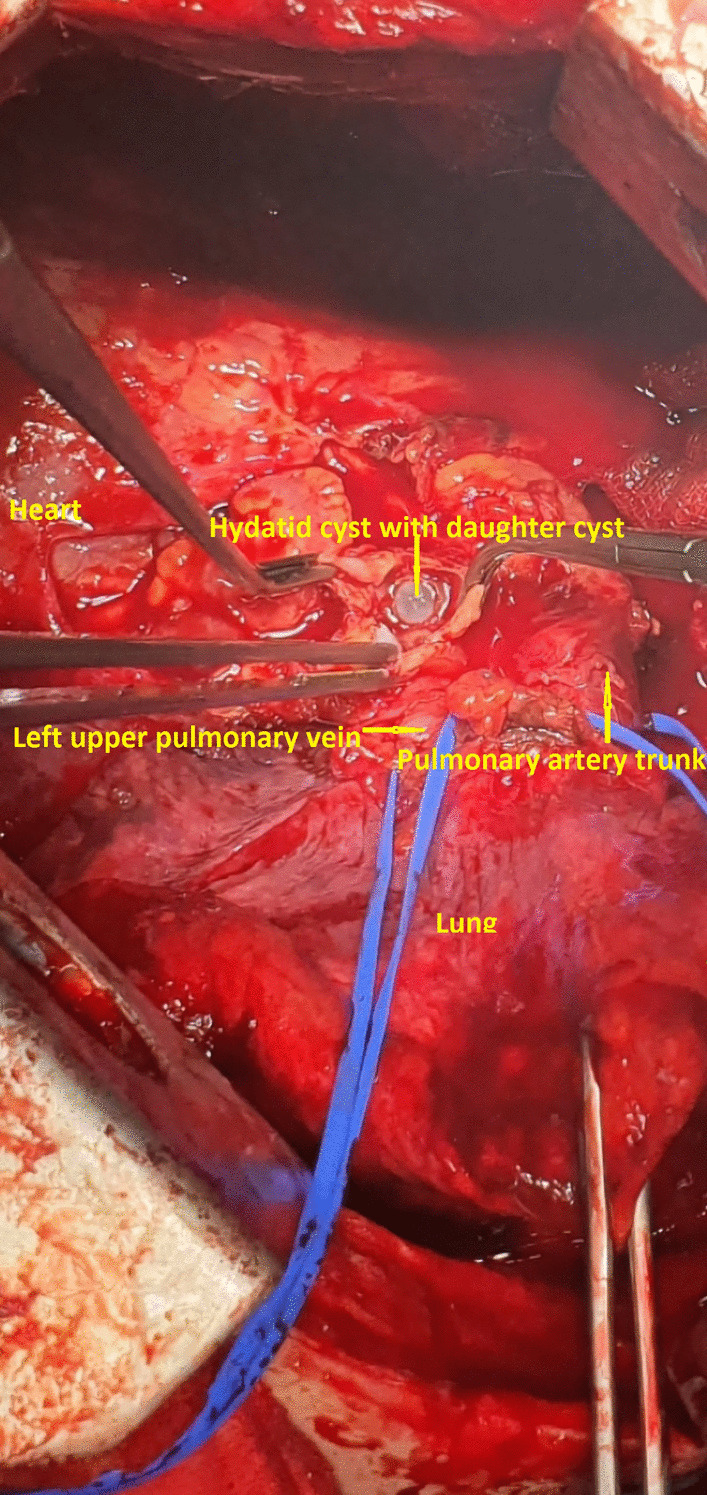
Intrapericardial hydatid cyst and daughter cysts within it, next to the left atrium and left upper pulmonary vein

After an intensive pre-operative work up and consultations and 7 days of premedication with albendazole, the patient underwent left anterolateral thoracotomy by thoracic surgeons. The opening of the pericardium was performed behind the phrenic nerve in order to follow from hilum of the lung the entrance of pulmonary veins intrapericardial. It was very difficult to localize the cystic mass once the pericardium was opened because of its retrocardiac location. In order to have a better access to the area where it was located, we decided to release the Botali duct. It took approximately 30 min to mobilize the cyst because it was densely adherent with the left atrium and in close proximity with left pulmonary veins compressing them. The cystic mass measured 5 cm in diameter and its content was completely removed after the surgical field was isolated with gauze impregnated with hypertonic solution (NaCl 10%). The cystic mass found was an echinococcal cyst with several daughter cysts measuring up to 10 mm within it. After removal of the daughter cysts and a partial resection of the pericyst, no involvement/penetration of the heart wall was found (Fig. 4). The remained cavity of the cyst was washed out with hypertonic solution (NaCl 10%) and left open. A wedge resection of the left upper lobe consolidation area was performed (the biopsy of specimen confirmed lung infarction, as was previously reported on biopsy performed by FNA). The pericardium was left opened and before the closure of the chest cavity, a chest drain was placed next to the area of pericardiotomy. Postoperatively the patient had a good recovery and was discharged from the hospital 5 days later in good conditions and was treated with three courses of 28 days with albendazole 400 mg × 2 tb/day.

## Discussion

Hydatid disease/Echinococcus cyst is a parasitic disease caused by an infection with the larvae of the tapeworm *Echinococcus granulosus* [1]. The most frequently involved site in the heart is the myocardium, while pericardial involvement occurs in 2–10% of cardiac echinococcosis cases and 0.5–2% of all hydatidosis cases [2–7]. Pericardial involvement of hydatid cysts may be caused by systemic circulation, which results from fissuring of hydatid cysts from the liver or lung, transdiaphragmatic dissemination or lymphatic circulation [2–5].

Patients with pericardial hydatid cysts may remain asymptomatic until echinococcal cysts cause pressure mass effects on surrounding structures. Presenting symptoms of uncomplicated pericardial hydatid cysts include chest pain due to stretching of the pericardium and/or compression of coronary vessels and dyspnea [2–5].

The diagnosis of hydatidosis is based on immunodiagnostic methods along with radiological and ultrasound examinations [1–8].

In our case, the antibody titer test was negative. False negative results in human hydatidosis may range from 3 to 5% of hydatid patients up to 35–40% in hyperendemic areas, as in the case of our country [8].

Although pericardial hydatid cysts can be detected by transthoracic echocardiography, cross-sectional imaging studies such as computed tomography (CT) and/or MRI are usually necessary to identify before the surgery the cardiac anatomy, disease location and cyst size [2–7].

CT is a better imaging technique for revealing small calcifications, which may be a helpful imaging finding in the diagnosis of a hydatid cyst. Our case had no calcifications and no other cysts in the liver or in the other structures. The relationship of hydatid cysts with surrounding structures can be seen by MRI [2–4]. Hydatid cysts present as a hypointense mass on T1-weighted MRI scans (T1-weighted) images and hyperintense mass on T2-weighted MRI scans (T2-weighted) images. A hypointense rim around the mass on T2-weighted scans represents the pericyst. Daughter cysts are multiple cystic structures attached to the internal wall of the cyst. In our case, CT showed a hypodense mass characteristic of hydatid cysts with multiple daughter cysts inside. On MRI, a multicameral cystic mass was seen with no cardiac involvement/penetration infiltration after contrast medium, with differential diagnosis of a cavernous lymphangioma vs hydatid cyst. On transesophageal ultrasound, a multi-lobulated extracardiac mass adjacent to the left atrium was seen, with mass effect and probably thrombus in the left pulmonary vein (Gharbi type II) [9].

Treatment of pericardial hydatid cysts can be accomplished with surgical excision of the cystic lesion. Medical treatment (e.g., albendazole and mebendazole) is complimentary for disseminated cases and for prophylaxis [2–7].

Research has shown that 73–75% of patients respond to medical management to some extent, but the reported cure rates are only 25–30%, and this strategy is a long and tedious process involving considerable risks [1].

In our case, considering the rare position of the cyst (retrocardiac) and the fact that the cyst did not enter the intracardiac site, the surgery was performed through left anterolateral thoracotomy by thoracic surgeons after 7 days of premedication with albendazole. It was necessary to release the Botalli duct after opening the pericardium to better expose the cystic lesion and all the structures adjacent to it. The surgery was performed in cardiac operation suite/room theatre with the availability of cardiac surgeons for any intraoperative complications.

There are a lot of cardiac surgeons that would prefer to perform the surgery in cases like that through the sternotomy, but in our case the cardiac surgeons were not familiar with the surgery in the area that the cystic mass was localized.

Regarding the premedication with albendazole prior to surgery there have been recent studies that emphasise the role of albendazole in cases of rupture of large cysts of the lungs [10]. In our case the location of the pathology was intrapericardial, it was not a big cyst but a cystic mass full of daughter cysts (hydatidosis) and the premedication was necessary to prevent the dissemination.

## Conclusion

Pericardial echinococcosis is a very rare pathology in which a high expertise multidisciplinary approach is required. The mass effect caused by the cyst can lead to complications, such as in our case where the pulmonary vein was compressed, causing a pulmonary infarction. The values of radiology and transesophageal ultrasound are very important in the diagnosis. Surgery in these cases is always recommended, but still the surgical approach is questionable. In cases such as ours, we recommend anterolateral thoracotomy.

## Data Availability

Data sharing is not applicable to this article, as no datasets were generated or analyzed during the current study.

## Abbreviations

CT: Computed tomography
CECT: Contrast-enhanced CT
FNA: Fine needle aspiration
MRI: Magnetic resonance imaging
T1-weighted: T1-weighted MRI scans
T2-weighted: T2-weighted MRI scans
